# Paeonol protects endotoxin-induced acute kidney injury: potential mechanism of inhibiting TLR4-NF-κB signal pathway

**DOI:** 10.18632/oncotarget.8347

**Published:** 2016-03-25

**Authors:** Hua-Ying Fan, Dong Qi, Chen Yu, Feng Zhao, Tao Liu, Zuo-Kai Zhang, Ming-Yan Yang, Lei-Ming Zhang, Da-Quan Chen, Yuan Du

**Affiliations:** ^1^ School of Pharmacy, Key Laboratory of Molecular Pharmacology and Drug Evaluation (Yantai University), Ministry of Education, Collaborative Innovation Center of Advanced Drug Delivery System and Biotech Drugs in Universities of Shandong, Yantai University, Yantai, P.R. China; ^2^ Department of Nephrology, Yu-Huang-Ding Hospital/Qingdao University, Yantai, P.R. China; ^3^ School of Pharmacy, Binzhou Medical University, Yantai, P.R. China; ^4^ Center for Reproductive Medicine, Tai'an Central Hospital, Tai'an, P.R. China

**Keywords:** paeonol, acute kidney injury, sepsis, TLR4, NF-κB signal pathway

## Abstract

**Study design and methods:**

In order to determine the therapeutic effect and mechanism of paeonol on acute kidney injury induced by endotoxin, an acute kidney injury model was established by intraperitoneal administration of lipopolysaccharide in mice *in vivo* and on LPS-induced dendritic cells *in vitro*. Renal tissues were used for histologic examination. Concentrations of blood urea nitrogen and serum creatinine were detected, inflammatory cytokines were determined by ELISA. The relative proteins' expression of TLR4-NF-κB signal pathway was assessed by Western blot, the localization and expression of phospho-NF-κB p65 in kidney was monitored by immunohistochemistry.

**Results:**

Treatment of paeonol successfully cuts histopathological scores and dilutes the concentrations of blood urea nitrogen and serum creatinine as index of renal injury severity. In addition, paeonol reduces pro-inflammatory cytokines and increases anti-inflammatory cytokines stimulated by LPS in a dose-dependent manner. Paeonol also inhibits the expression of phosphorylated NF-κB p65, IκBα and IKKβ, and restrains NF-κB p65 DNA-binding activity. Paeonol treatment also attenuates the effects of LPS on dendritic cells, with significant inhibition of pro-inflammatory cytokines release, then TLR4 expression and NF-κB signal pathway have been suppressed.

**Conclusions:**

These results indicated that paeonol has protective effects on endotoxin-induced kidney injury. The mechanisms underlying such effects are associated with its successfully attenuate inflammatory and suppresses TLR4 and NF-κB signal pathway. Therefore, paeonol has great potential to be a novel and natural product agent for treating AKI or septic-AKI.

## INTRODUCTION

Acute kidney injury (AKI), also as acute renal failure (ARF), is clinically defined as an abrupt and reversible deterioration of glomerular and tubular function. AKI is the common clinical manifestation of multiple mechanisms and causes of injury, which ranges from septic shock to crescentic glomerulonephritis, from rhabdomyolysis to drug-induced interstitial nephropathy, from hepatorenal syndrome to obstructive nephropathy [1]. AKI is increasing in incidence associated with considerable morbidity and mortality. The incidence rate of AKI in hospitalized patients is about 7% and AKI rates in intensive care units (ICUs) have been reported to be up to 22%–67% and carries 50–80% mortality [2]. Approximately 2 million people in the world will die yearly of acute kidney injury (AKI), a disease without valid treatment exists [3, 4].

Currently, supportive renal replacement therapy is the only treatment option available for AKI, but the observed mortality for the more serious end of the spectrum of AKI remains high in the range of 50–60% when supported by RRT [5]. This observed mortality has hardly changed in recent decades despite advances in renal support technology [6].

Although AKI is often a complex multifactorial syndrome, AKI is most commonly caused by sepsis. The kidney is the most sensitive target organ of sepsis [7]. The mortality in hospitalized patients with AKI complicating severe sepsis is more than 70% compared with 39% in patients with non-septic AKI [8, 9].

Sepsis is a serious clinical condition characterized by overwhelming infection that results from a complex interaction of pathogen and host harmful inflammatory response to infection [10]. The endotoxin is one of the major triggers of inflammatory responses in sepsis, as it is known to product and releases a large number of inflammatory cytokines that activate potent immune response through the activation of Toll-like receptor 4 (TLR4) [11]. TLR4 activation could further trigger transcription of nuclear factor-κB (NF-κB), which downregulates inflammatory genes, upregulates anti-inflammatory genes, and induces the apoptosis of leukocytes. [12]. Therefore, suppressing abnormal immune responses through TLR4-NF-κB pathway might prevent AKI and improve the clinical outcome in sepsis.

**Figure 1:**
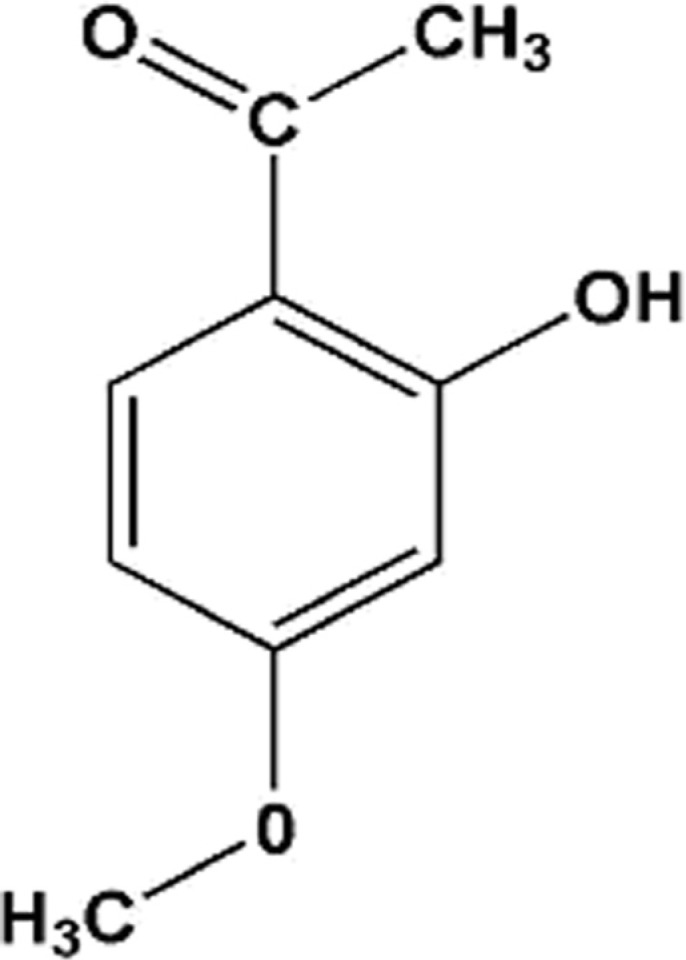
The chemical structure of paeonol (C_9_H_10_O_3_, molecular weight = 166.18).

Paeonol (2-hydroxy-4-methoxyacetophenone; C_9_H_10_O_3_, Figure 1), is the main active phenolic compound isolated from the root bark of herbal plant *Paeonia moutan Sims* (*Paeonia suffruticosa Andrews, Paeoniaceae*), which has been widely used as traditional Chinese medicine for centuries to treat various diseases including rheumatoid arthritis, systemic lupus erythematosus, hepatitis, dysmenorrhea, muscle cramping and spasms [13, 14]. In recent years, increasing attentions have been paid towards the potential various pharmacological properties of paeonol, which possesses various pharmacological activities including sedation, antipyresis, analgesic, anti-tumor, anti-oxidation, anti-inflammation and immunoregulation and so on [15].

Paeonol has also been reported to inhibit osteoclastogenesis by inhibiting ERK, p38 and NF-κB pathway [16]. It can prevent ox-LDL and TNF-α from inducing monocyte adhension to vascular endothelial cells [17, 18]. Research also indicates that paeonol can inhibit the expression of cell-surface adhesion molecules [19], proinflammatory cytokines such as TNF-α and IL-1β [20, 21], iNOS-mediated NO [22] and reactive oxygen species production [20, 22]. What's more, paeonol inhibits the generation of proinflammatory cytokine and increases the production of IL-10 in carrageenan-evoked thermal hyperalgesia rats [23]. However, no available study has demonstrated the effects of paeonol treatment on AKI. Therefore, this study is performed to investigate the effect of paeonol on LPS-induced AKI and the mechanisms responsible for it to provide more possibility and rationality for its application in treatment of AKI.

## RESULTS

### Effects of paeonol on LPS-induced renal injury

**Figure 2:**
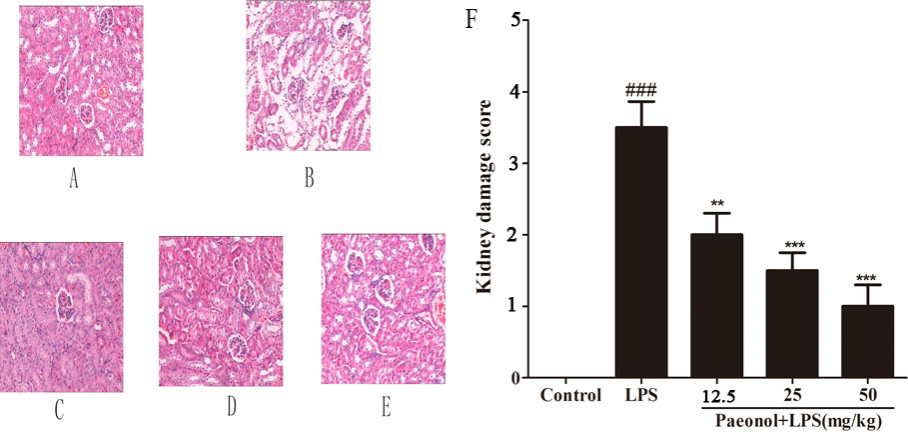
Effect of paeonol on kidney injury after LPS administration Representative histological changes in kidneys obtained from mice of different groups (**A**) Control group; (**B**) LPS group; (**C**) Paeonol (12.5 mg/kg) + LPS group; (**D**) Paeonol (25 mg/kg) + LPS group; (**E**) Paeonol (50 mg/kg) + LPS group. The sections shown were harvested 12 h after LPS injection and stained with H & E. Magnification: ×400. (**F**) Pathological score of representative kidney samples of each group. Data are represented as mean ± SD of 5 animals of each group and we repeated the experiments three times. ^###^*p* < 0.001 versus control, **p < 0.01 and ***p < 0.001 versus LPS group.

Our histological examination has shown that glomerular membrane and the structure of epithelial cell are intact and clear in control group. In the LPS-induced mice renal tissues, histological examination has shown severe lesions, with wide degeneration of renal tubular epithelial cells, glomerular atrophy and dilation of renal capsule cavity. In addition, severe hyperemia of renal tubule interstitial and cell debris concentration can be seen in renal tubule lumens. The histopathological injury score has been significantly elevated as shown in Figure 2. However, treatment of animals with paeonol has shown significant effect at all dose levels. The lesion in mice treated with paeonol was alleviated when compared with that in LPS-treated alone group, with less injured area and less extent of congestion or edema.

**Figure 3:**
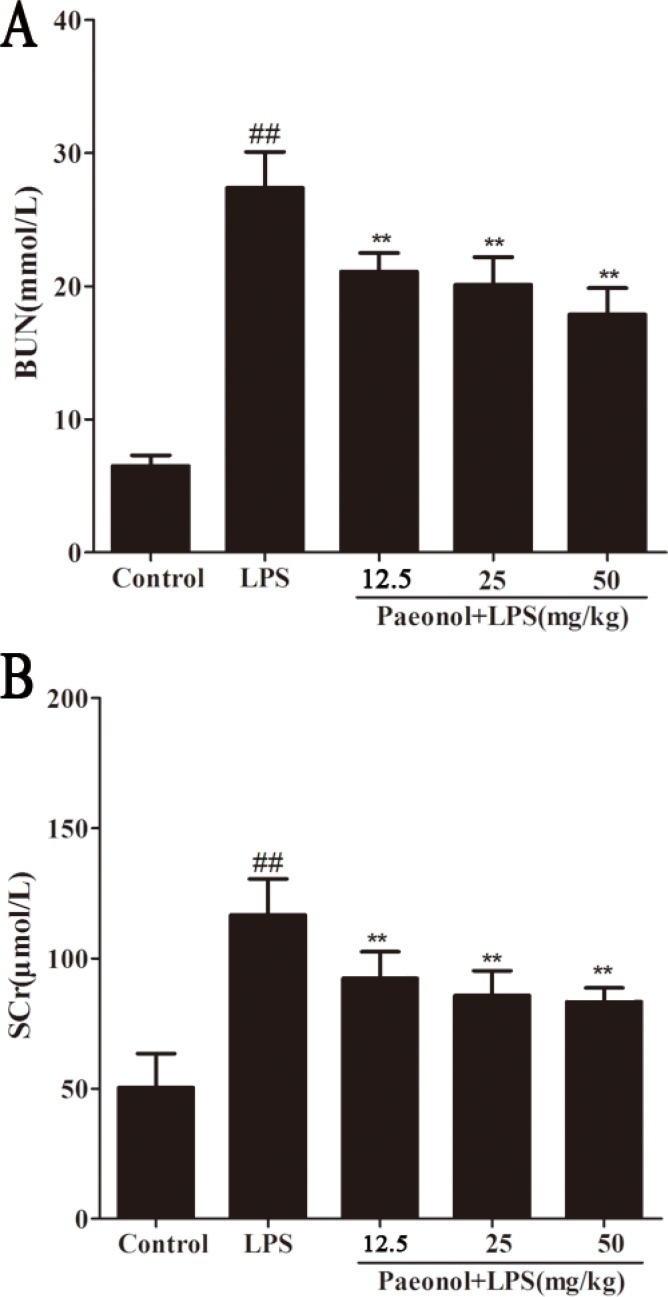
Effects of paeonol on serum BUN and SCr Data are represented as mean ± SD of 10 animals of each group and we repeated the experiments three times. **p < 0.01, ***p < 0.001 compared to LPS group; ^##^*P* < 0.01 compared to control group.

BUN and SCr as important index of renal injury severity were used for the assessment of renal function. The changes of BUN and SCr in serum were potentially associated with kidney injury. The results have shown that mice treated with LPS showed significantly elevated levels of BUN and SCr compared with those in the control group. Pretreatment with paeonol (12.5, 25, 50 mg/kg) decreased the level of BUN and SCr induced by LPS (Figure 3).

### Effects of paeonol on cells viability

**Figure 4:**
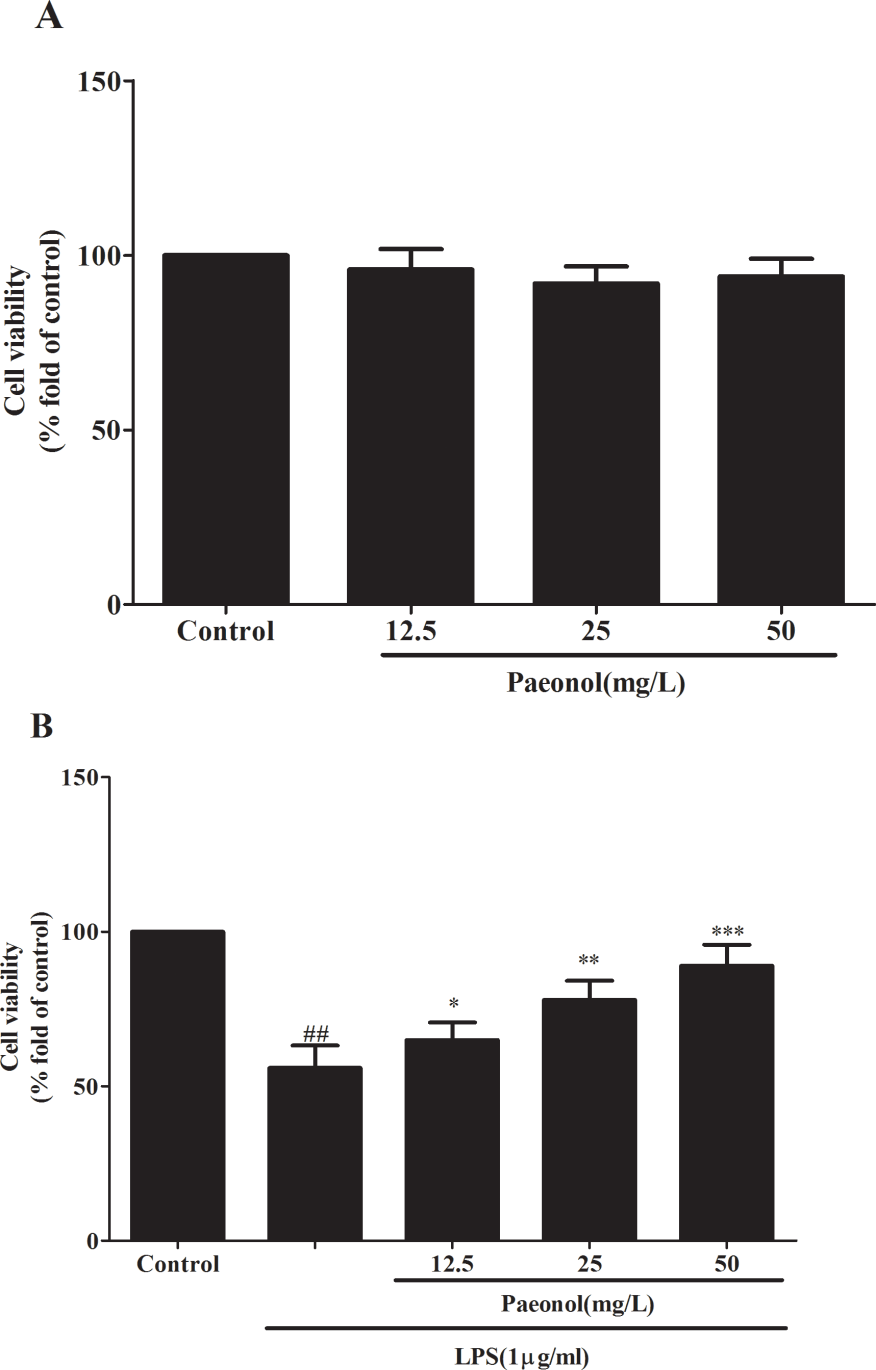
The effects of paeonol on DCs viability were tested by MTT assay (**A**) Effect of osthole on DCs proliferation in normal condition by MTT assay. (**B**) Effect of osthole on LPS-induced DCs proliferation by MTT assay. Results are expressed as percentage of viable cells when compared with control groups. we repeated the experiments three times. ^##^*p* < 0.01 vs. untreated group, *p < 0.05, **p < 0.01 vs. LPS alone.

The proliferation of DCs was evaluated by using MTT assay. As shown in Figure 4A, DCs viability was not significantly altered by paeonol treatment under normal condition. In contrast, LPS could markedly inhibit DCs proliferation, which was significantly enhanced by paeonol in a concentration dependent manner (Figure 4B). It is revealed that paeonol did not exhibit cytotoxicity against DCs and could enhance cell survival induced by LPS.

### Effects of paeonol on LPS-induced inflammatory responses

**Figure 5:**
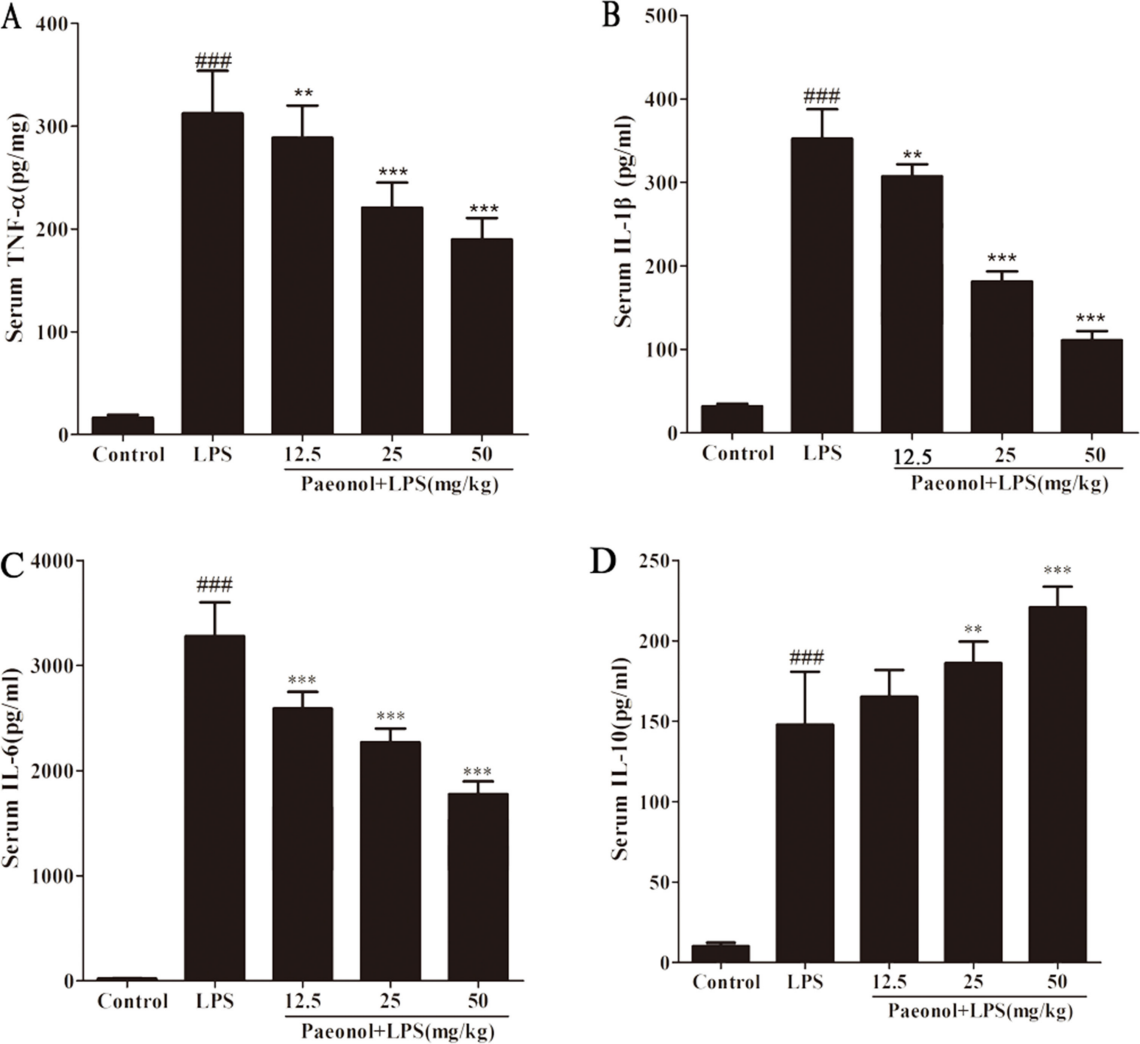
Effects of paeonol on the production of inflammatory cytokinesin in serum from mice after LPS challenge Quantitation of TNF-α (**A**), IL-1β (**B**), IL-6 (**C**) and IL-10 (**D**) in serum was performed by ELISA. Data are represented as mean ± SD of 10 animals of each group and we repeated the experiments three times. *p < 0.05 and **p < 0.01, ***p < 0.001 compared to LPS group; ###*p* < 0.001 compared to control group.

**Figure 6:**
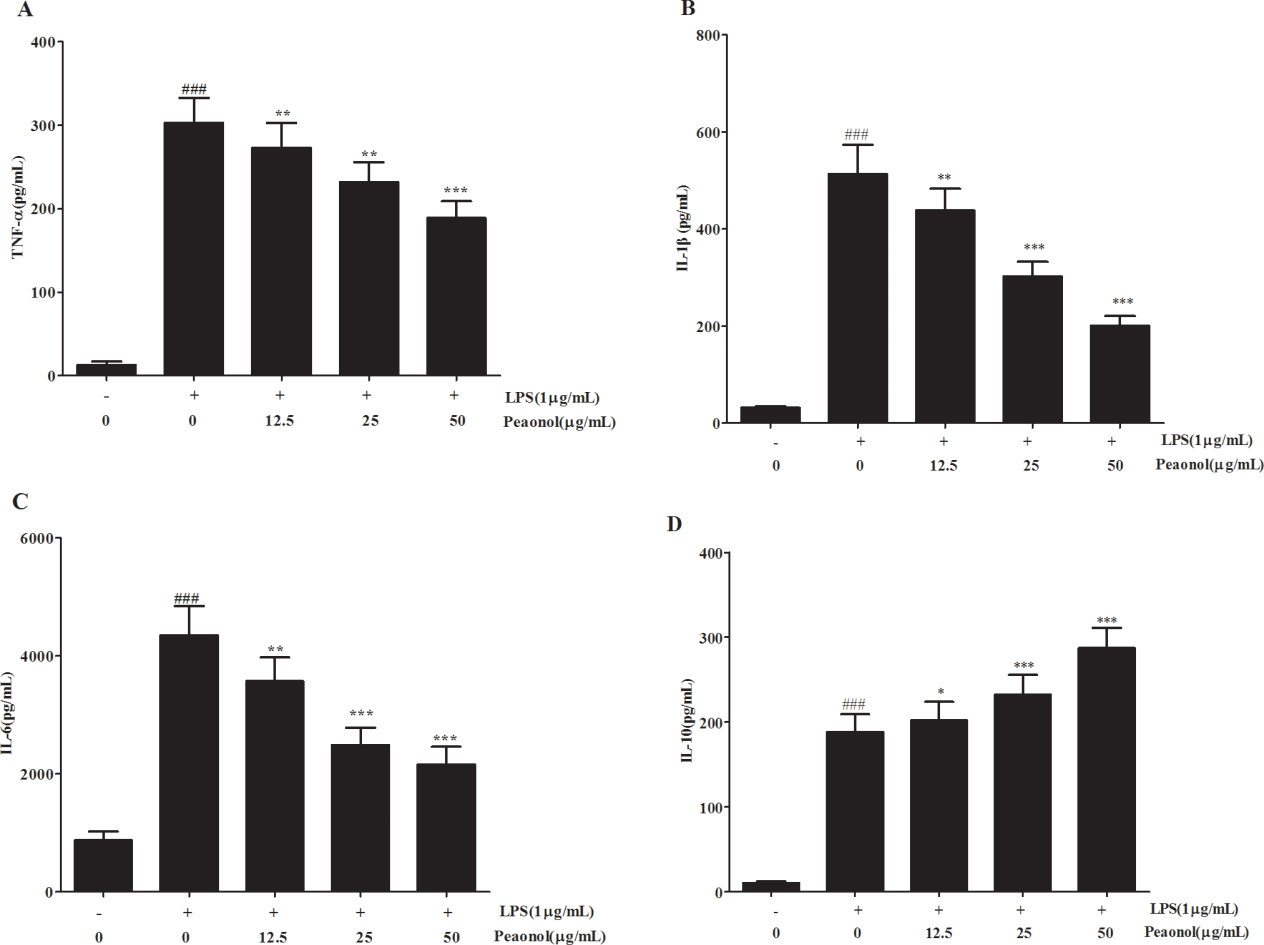
Effects of paeonol on the production of inflammatory cytokinesin by LPS-induced DCs Quantitation of TNF-α (**A**), IL-1β (**B**), IL-6 (**C**) and IL-10 (**D**) in cultural supernatants was performed by ELISA. Data are represented as mean ± SD of 10 animals of each group and we repeated the experiments three times. **p < 0.01 compared to LPS group; ^##^*p* <0.01, ^###^*p* < 0.001 compared to control group.

For a better understanding of potential mechanisms by which paeonol exerted a protective effect on renal function, we also analyzed the levels of inflammatory cytokines induced by LPS *in vivo* and *in vitro*. As shown in Figure 5, LPS-treated mice had significantly increased levels of TNF-α, IL-1β and IL-6 at 12 h after lipopolysaccharide injection, while paeonol pretreatment significantly reduced TNF-α, IL-1β and IL-6 production by LPS treatment in a dose-dependent manner. IL-10 in kidney was also measured to evaluate the potential anti-inflammatory response. IL-10 greatly increased in the paeonol treatment group compared with the LPS group. The results are consistent with that obtained from *in vitro* DC (Figure 6). These data suggested that paeonol could attenuate LPS-induced inflammatory responses by regulating the production of inflammatory cytokines.

### Effect of paeonol on TLR4 and NF-κB signal pathway

TLR4-NF-κB signal pathway plays a critical role in the inflammation cytokine production. To investigate the mechanism by which paeonol inhibits LPS-induced production of inflammatory cytokines, we assessed the expression of TLR4 and the related proteins expression in NF-κB signal pathway *in vivo* and *in vitro*. Treatment of mice with a single dose of LPS caused a significant augmentation in phosphorylation level of IKKβ, IκBα and NF-κB p65 as compared to the control group treated with saline. This indicates that NF-κB activity was increased. Administration of paeonol down-regulated the expression of phosphorylated NF-κB p65, IKKβ and IκBα (Figure 8). These phenomena were confirmed in the DCs culture system *in vitro* and the expression of TLR4 protein was also significantly inhibited by paeonol (Figure 9).

**Figure 7:**
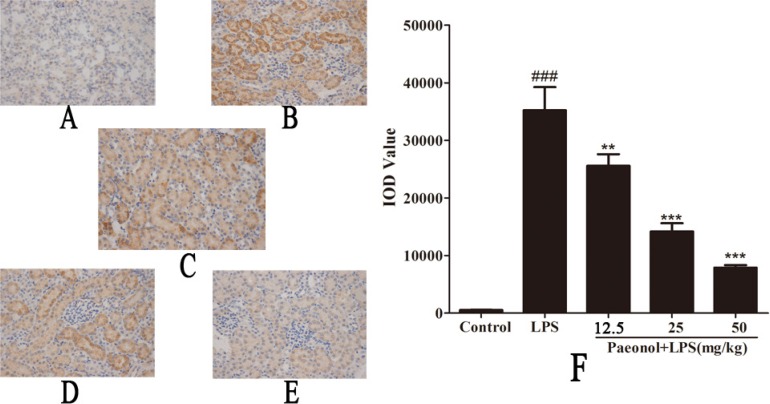
Effect of paeonol on phospho-NF-κB p65 localization and expression in AKI by immunohistochemistry (magnification×400) (**A**) Control group; (**B**) LPS group; (**C**) Paeonol (12.5 mg/kg) + LPS group; (**D**) Paeonol (25 mg/kg) + LPS group. (**E**) Paeonol (50 mg/kg) + LPS group. (**F**) IOD values of phospho-NF-κB p65 staining. Data are represented as mean ± SD of 5 animals of each group and we repeated the experiments three times., ^###^*p* < 0.001 versus control, **p < 0.01, ***P < 0.001 versus LPS group.

**Figure 8:**
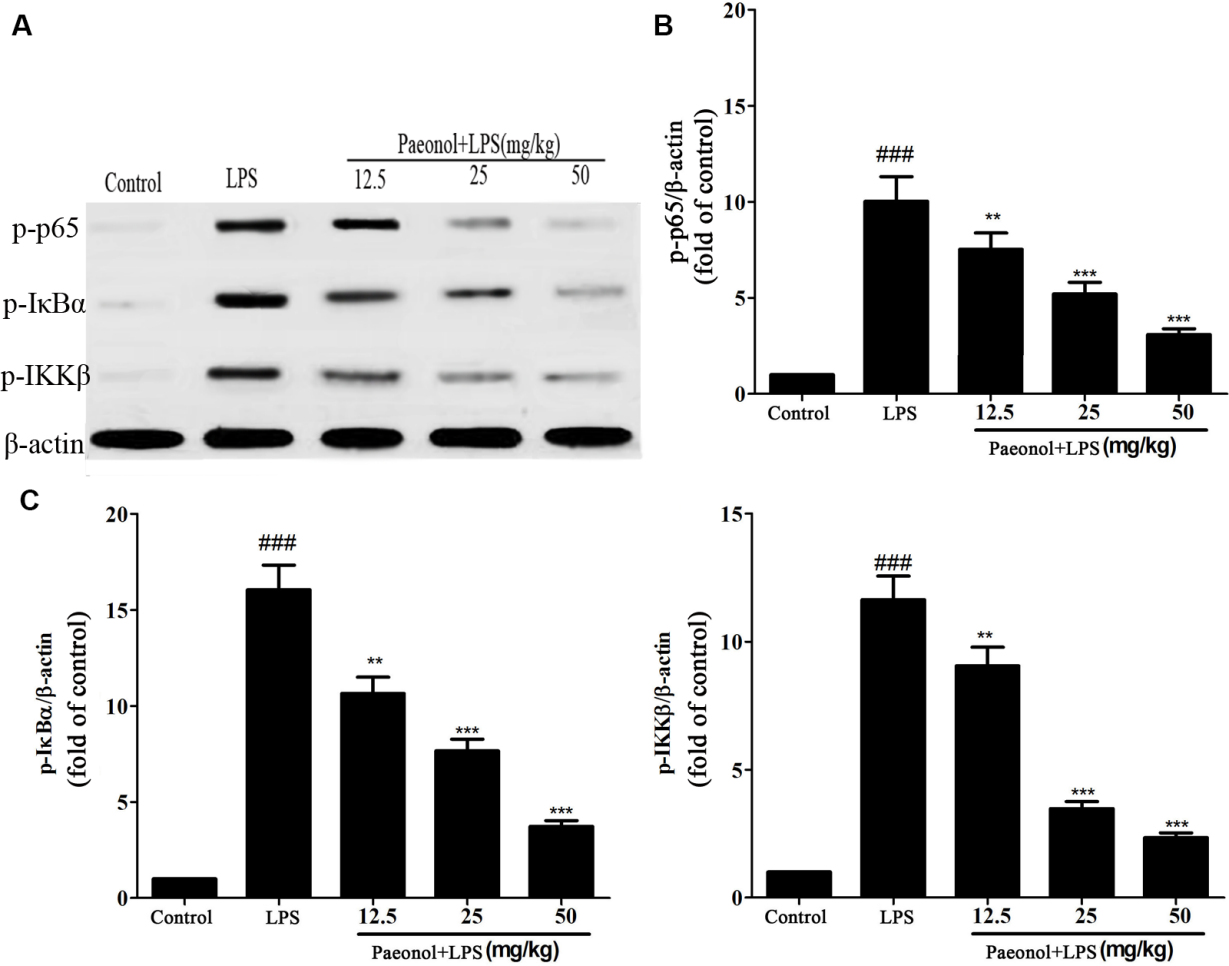
Effects of paeonol on the activation of the NF-κB signalling pathway in LPS-induced AKI BALB/c mice were treated with vehicle or paeonol for 7 days. After the last of administration, all mice except the control group received a single intraperitoneal injection of 10 mg/kg of LPS to induce AKI. Twelve hours after the LPS injection, kidney tissues were collected to measure the phosphorylation of IKKβ (**A**), IκBα (**B**) and p65 (**C**) by Western blot. β-actin was used as a standard control. Results are expressed as fold increase over control group. All data represent the means ± SD from three separate experiments. **p < 0.01, ***p < 0.001 compared to LPS group; ^##^*p* < 0.01 and ^###^*p* < 0.001 compared to control group.

**Figure 9:**
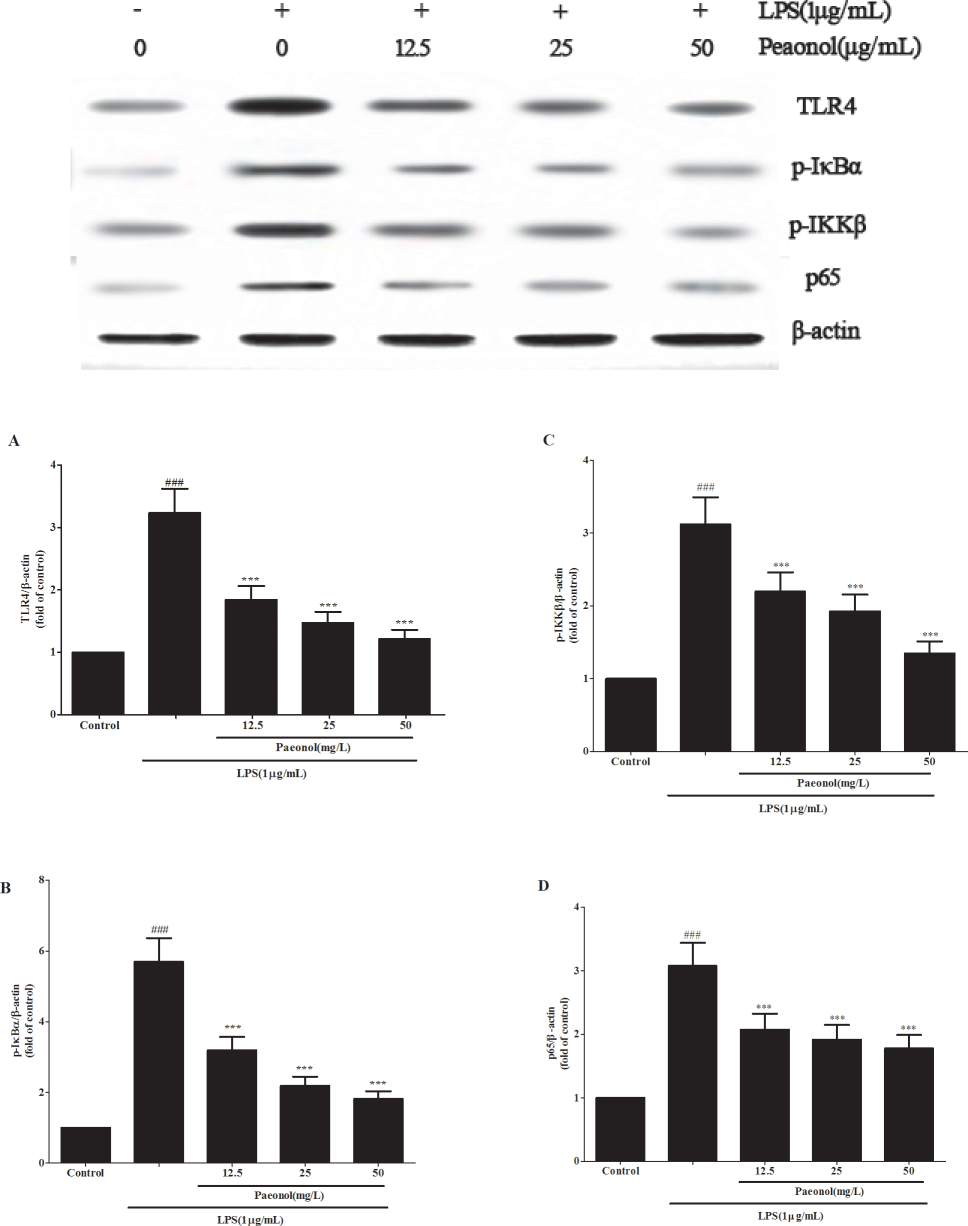
Paeonol modulates LPS-stimulated DCs by TLR4-NF-κB signaling DCs were incubated in presence or absence of different concentrations of paeonol (12.5, 25, 50 mg/L) for 24 h, then incubated with or without 1 μg/mL LPS for another 24 h. The expression levels of TLR4 (**A**), the phosphorylation of IKKβ (**B**), IκBα (**C**) and p65 (**D**) in cell lysates were determined by Western blot. β-actin was used as a standard control. Results are expressed as fold increase over control group. All data represent the means ± SD from three separate experiments. **p < 0.01, ***p < 0.001 compared to LPS group; ^##^*p* < 0.01 and ^###^*p* < 0.001 compared to control group.

**Figure 10:**
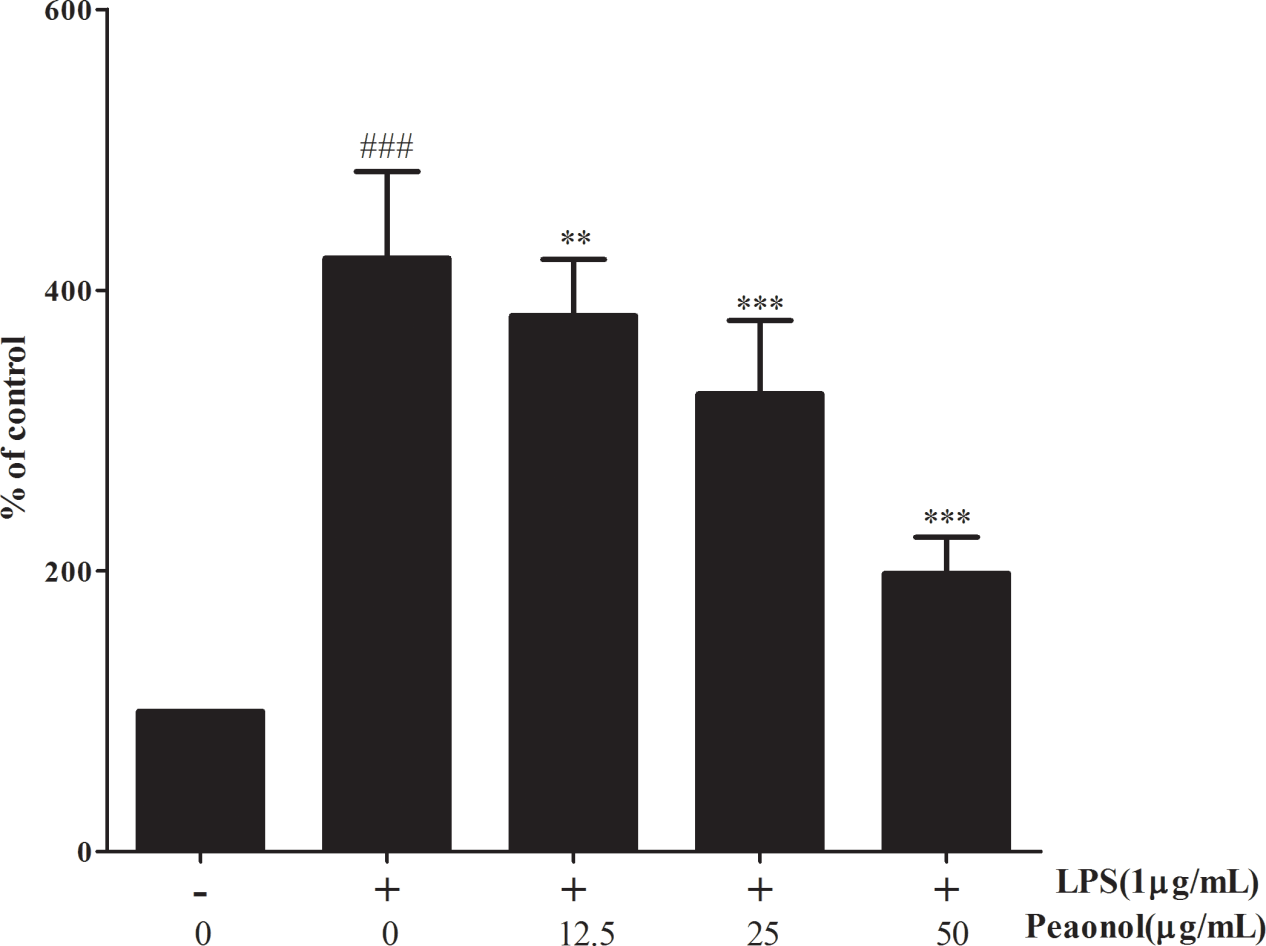
The effect of paeonol on the DNA-binding activity of NF-κB in DCs The DNA-binding activity of NF-κB in nuclear extracts was assessed using the ELISA-based NF-κB transcription factor assay kit 24 h after stimulation in RAW 264.7 cells. Untreated group is set as 100%. Results are expressed as fold increase over untreated group. Data are represented as mean ± SD of three independent experiments. ^##^*p* < 0.01 vs. untreated group, **p < 0.01 vs. LPS alone.

Furthermore, Immunostaining for phosphor-NF-κB p65 was measured to demonstrate its localization in kidney sections. As shown in Figure 7, immunostaining for phosphorylated NF-κB p65 demonstrated its expression and localization in kidney sections. Staining for phosphorylated NF-κB p65 in nuclei and cytoplasm of proximal convoluted tubule and renal glomerulus was more pronounced in LPS-induced group mice than in control mice. Paeonol administration attenuated the NF-κB p65 staining. Paeonol could affect the DNA binding activities of NF-κB subunits by using the ELISA-based NF-κB transcription factor assay kit. LPS treatment strongly promoted the binding of NF-κB p65 to DNA (Figure 10). Whereas paeonol treatment mitigated LPS-induced NF-κB p65 binding activity dose dependently. Our finding suggests that paeonol may reduce NF-κB signaling pathway activation via the inhibition of the nuclear translocation and DNA-binding activity by regulating phosphorylation of IKKβ and IκBα.

## DISCUSSION

Sepsis has been regarded as the most common cause of AKI in intensive care units. In addition, the combination of sepsis and AKI is related to a very high mortality rate [24]. Considering the high incidence and related morbidity and mortality of sepsis associated with AKI, there is an urgent medical need to investigate novel pharmacological interventions to treat or prevent AKI. Experimental endotoxemia induced by LPS is the most frequently employed model to study septic AKI. LPS (lipopolysaccharide), an endotoxin, is a major component of the outer membrane of Gram-negative bacteria, which is considered the main triggers of inflammatory responses in sepsis [25]. This model can produce consistent renal tissue damage which is similar to that observed in humans [26, 27, 28]. The goal of the current study was not only to investigate paeonol as a potential therapeutic approach for LPS induced AKI, but also to uncover the mechanism of sepsis induced AKI

In the present study, murine AKI model has been successfully established by treating BALB/c mice with a single intraperitoneal injection of 10 mg/kg of LPS according to the previous study [29, 30]. This model of endotoxemia presented a substantial kidney injury with obvious changes of histopathology and serum biochemical index of renal injury. Histopathology examination has showed that the glomerular structure is destroyed, renal tubular epithelial cell degenerated and there were severe intracellular edema and congestion within renal tubule and renal interstitium. In addition the level of BUN and SCr as an index of renal injury is also higher. Treatment with paeonol, however, could attenuate the changes of histopathology and reduce the increase of BUN and SCr. It suggests that paeonol could attenuate kidney damage in LPS-induced AKI.

Although the pathogenesis of AKI during septic shock is not entirely clear, excessive inflammation response plays an important role [31]. Dysregulated inflammatory cytokines release triggers the pathophysiological abnormities of sepsis and multi-system organ failure [32]. To explore the underlying mechanisms of beneficial effect on septic-AKI, the levels of inflammatory cytokines were measured. We demonstrated that paeonol attenuated proinflammatory cytokines and increased anti-inflammatory cytokines IL-10 level dose-dependently following LPS administration both *in vivo* and *in vitro*. IL-10 is a pleiotropic cytokine possessing both anti-inflammatory and immunosuppression properties [33]. In the acute phase of sepsis, endogenous IL-10 production and exogenous administration can reduce the magnitude of the inflammation [34]. It is inferred that the protection of paeonol against septic-AKI may be related to its modulation of the immune response by regulating inflammatory cytokines.

Dendritic cells (DCs) are professional antigen presenting cells (APCs) able to link the innate and the adaptive immune responses [35]. Toll-like receptor 4 (TLR4) is a cellular receptor for LPS expressing in the surface of DCs. LPS binds to TLR4, leading to activation of downstream pathways that can activate nuclear factor NF-κB [36]. It is well known that genes encoding of many pro-inflammatory cytokines are to be under the control of NF-κB transcription factors [37]. NF-κB is one of the most important and widely used transcription factors and is involved in the regulation of gene expression in LPS-induced inflammation response during kidney injury and pathophysiology of sepsis [38]. NF-κB is kept in an inactive form in the cytoplasm by interacting with inhibitor of NF-κB proteins (IκB), of which the best-studied and major one is IκBα. IκB is phosphorylated by IκB kinases (IKKs) and ubiquitinated, leading to its degradation by the proteasome. The activation of NF-κB is regulated by IκB and IKKs, which can be activated by several stimuli such as lipopolysaccharide, pro-inflammatory cytokines and virus. These stimulus can active the IKKs complex [39]. IKKs can phosphorylate the inhibitory IκBα protein and this phosphorylation results in the dissociation of IκBα from NF-κB. Then the uncovered nuclear localization signals will cause the activation of NF-κB proteins, which liberate NF-κB to the nucleus and activate NF-κB target genes [40]. Along with the NF-κB activation, the expression levels of several cytokines (IL-8, IL-6 and TNF-α) and adhesion molecules all increased. Since NF-κB is the key regulator in the AKI inflammatory response, it becomes the most attractive target in treating septic AKI [41]. In order to determine whether paeonol affect NF-κB activity, we planned to investigate the key molecules that regulate TLR4-NF-κB signaling pathway. Our results showed that treatment with paeonol inhibited the activation of IKK, resulting in the decreased phosphorylation and subsequently less degradation of IκB, which in turn leads to less activation and translocation of NF-κB. Furthermore, paeonol prevented TLR4 expression and DNA-binding activity of NF-κB p65 subunit by inhibiting the phosphorylation of IKKβ, IκBα and p65 in DCs.

In conclusion, we have provided the first evidence that pre-treatment of paeonol attenuates LPS-induced acute kidney injury in mice, so that it has a significant effect on the development and progression of septic AKI. The underlying mechanism of paeonol on anti-endotoxin kidney injury may be closely related with regulating the production of inflammatory cytokines and activation of the TLR4-NF-κB signal pathway through restraining the TLR4 expression and phosphorylation of the relative proteins in the NF-κB signal pathway and hindering DNA-binding activity of NF-κB. This evidence suggests that paeonol has a potential application to treat endotoxemia-associated acute kidney injury.

## MATERIALS AND METHODS

### Reagents and animals

Paeonol (purity = 98%, HPLC) were purchased from National Institute for the Control of Pharmaceutical and Biological Products (Beijing, China). Lipopolysaccharide (LPS) from Escherichia coli 055:B5 was obtained from Sigma-Aldrich Chemical Co. (USA). Mouse TNF-α and IL-1β ELISA Kits were purchased from Shanghai Chuanxiang Biotechnology Ltd Co. (Shanghai, China), IL-10 ELISA Kit was obtained from the Bender MedSystems (Vienna, Austria), IL-6 ELISA Kit was purchased from eBioscience Inc.(San Diego, California, USA). Antibodies used in this study were anti-TLR4 antibody (ab30667, Abcam), anti-NF-κB p65 (ab86299, Abcam), anti-IKKβ (pTyr-199, ab59195, Abcam), anti-p-IκBα (Ser-36, ab133462, Abcam). Goat anti-rabbit IgG were purchased from Boster Biotechnology (Wuhan, Hubei province, China). Blood Urea Nitrogen (BUN) and Serum Creatinine Determination (SCr) assay kit reagents were supplied by were purchased from the Institute of Jiancheng Bioengineering (Nanjing, China). RPMI medium and fetal bovine serum (FBS) were products of Gibco Corporation (USA). The other reagents were all purchased from Sigma-Aldrich Chemical Co. (USA).

Eight-week-old BALB/c mice were purchased from Vital River Laboratory Animal Technology Co. Ltd. (Certificate No.: 0247652). All animals were acclimated for at least 1 week at a temperature of 24 ± 1°C and humidity of 55 ± 5%. The animals were maintained with free access to standard diet and tap water.

### Ethics statement

All the animal experiments in our study were performed in accordance with the Guide for the Care and Use of Laboratory Animals, formulated by the National Institutes of Health, USA, and approved by the Office of Experimental Animal Management Committee of Shandong Province, China and local Animal Ethical Committee (approved dates on 3/5/2013)

### Cell

Dendritic cells (DCs) were generated from the bone marrow of mice as previously described [42]. Bone marrow cells were harvested from the femur and tibia of mice. Cells were washed once with medium and re-suspended in RPMI medium containing granulocyte macrophage colony stimulating factor (GM-CSF) for 7–9 d. DC yield was monitored with flow cytometry after anti-CD11c staining.

### Experimental protocol

The mice were randomly divided into the following groups (10 in each group): control group, LPS-induced group and LPS + paeonol (12.5, 25, 50 mg/kg) group. Paeonol was dissolved in 5% carboxymethylcellulose sodium as vehicle. The mice in the control and LPS-induced group were fed with 5% carboxymethylcellulose sodium, while LPS + paeonol group were intragastrically (i.g.) given 12.5, 25 and 50 mg/kg paeonol respectively, once a day for 7 days. After the last of administration, all mice except the control group received a single intraperitoneal injection of 10 mg/kg of LPS to induce AKI. After twelve hours of the LPS injection, blood was collected by retroorbital venous plexus and processed to prepare serum. Both kidneys were harvested immediately following sacrifice. Right kidneys were processed for histopathology and immunohistochemistry and left kidneys were snap-frozen in liquid nitrogen for western blot assay.

### Histopathological examination

At the end of the experiments, the mice were dead and kidneys were removed and fixed in 4% paraformaldehyde for 24 h. The tissues were sealed up in paraffin and cut into 5 μm sections and then stained with hematoxylin and eosin for morphological examination. Renal tubular injury was assessed using a semiquantitative score in which the percentage of cortical tubules showing epithelial necrosis was assigned a score of either 0, none; 1, < 10%; 2, 10–25%; 3, 25–75%; or 4, > 75%. The semiquantitative score for kidney injury was calculated for each animal at least 10 fields by a blinded observer.

### Biochemical measurements

Blood samples were collected from the retroorbital venous plexus and centrifuged at 4°C for 10 min at 1400 × g in glass tubes; the serum was stored at −80°C in polystyrene tubes until use. The concentration of BUN and SCr was analyzed by using commercial kits reagents at the end of the experiment. The levels of TNF-α, IL-1β, IL-6 and IL-10 in serum from each group were determined by using mouse ELISA kits according to the manufacturer's instructions. The absorbance was measured using SpectraMax M2 Multi-Mode microplate reader.

### Immunohistochemical studies

For immunostainings, sections were deparaffinized, rehydrated in graded alcohols, and blocked by incubating in 0.3% H_2_O_2_ for 30 min. Antigen retrieval was performed by treating the slides in citrate buffer in a microwave oven for 10 min. The slides were incubated with primary antibodies of phosphorylated NF-κB p65 diluted to 1:200 in PBS for 16 h at 4°C. After a complete wash in phosphate buffered saline (PBS), the tissue slides were incubated with biotin-conjugated secondary antibodies for 1 h at 37°C and incubated with avidin-biotin peroxidase complex for 30 min at 37°C. The signal was detected using diaminobenzidine (DAB).

### MTT assay for cell viability

The 3-(4, 5-dimethyl-2-thiazolyl)-2, 5-diphenyl tetrazolium bromide (MTT) assay was used to indicate of cell proliferation. Cells were seeded at 10^4^ cells/well in 96-well plates with serum-free medium for 24 h incubation. Cells were incubated in presence or absence of different concentrations of paeonol (12.5, 25, 50 μg/mL) for 24 h, and then incubated with or without 1 μg/mL LPS for another 24 h. Then 20 μL of MTT (5 mg/mL) was added to each well and incubation continued at 37°C for additional 4 h. After removing the supernatant, 100 μL of DMSO was added to dissolve the reduced formazan. The absorbance at 570 nm wavelength was measured by using a microplate reader. The control group consisting of untreated cells was considered as 100% of viable cells. Results are expressed as percentage of viable cells when compared with control groups.

### Cytokine assays *in vitro*

DC were seeded in 12-well culture plates at the density of 1 × 10^6^ cells/mL and pretreated with various concentrations (12.5, 25, 50 μg/mL) of paeonol for 24 h, followed by LPS (1 μg/mL) for 24 h. The levels of TNF-α, IL-1β, IL-6 and IL-10 in the supernatants were determined by using commercial ELISA kits according to the manufacturer's instructions.

### Western blot analysis

Cells treated with different concentrations of paeonol followed by LPS treatment (1 μg/mL), were lysed and homogenized in lysis buffer immediately. The kidneys tissue samples were homogenized on ice followed by centrifugation at 12000 × g for 30s. The proteins were extracted according to instructions of total protein extraction kit. Protein concentrations were determined by BCA protein assay kit. The protein samples were separated by SDS-PAGE and were transferred to a PVDF membrane. The membrane was blocked with Tris buffered saline (TBS) containing 5% non-fat dry milk at room temperature for 2 h. For immunoblotting the membranes were incubated overnight at 4°C with antibodies directed against TLR4 (1:1000), NF-κ B p65 (1:5000), phospho (p)-(Tyr199)-IKKβ(1:1000), p-(Ser36)-IκBα (1:5000) and β-actin antibody (1:1000) used as loading controls. The secondary antibody (Horseradish peroxidase-conjugated anti-rabbit IgG antibody) was incubated at room temperature for 2 h. Protein was detected by using an enhanced chemiluminescence detection kit (Beyotime Institute of Biotechnology) and were scanned and quantified with Image J. The ratio for the protein examined was normalised against β-actin bands. Results were expressed as fold increase over control.

### Assay of NF-κB DNA-binding activity

DC were treated with different concentrations of paeonol or LPS (1 μg/mL) for 24 h. After extraction of the nuclear protein by using the nuclear extract kit, the DNA-binding activity of NF-κB p65 was assayed using the NF-κB p65 ELISA kit.

### Statistical analysis

All data were expressed as means ± SD. Statistical significance of differences between groups was determined by ANOVA. Differences were considered significant at *p* < 0.05.

## Abbreviations

AKI: acute kidney injury
ICU: intensive care unit
RRT: renal replacement therapy
NF-κB: nuclear factor-κappa B
LPS: Lipopolysaccharide
DCs: Dendritic cells
BUN: Blood Urea Nitrogen
SCr: Serum Creatinine
TNF: tumor necrosis factor
IL-1β: interleukin-1β
IL-6: interleukin-6
TLR4: Toll-like receptor 4
